# Linoleic Acid Inhibits the Release of *Leishmania donovani* Derived Microvesicles and Decreases Its Survival in Macrophages

**DOI:** 10.3389/fcimb.2020.00406

**Published:** 2020-08-07

**Authors:** Sheetal Saini, Ambak Kumar Rai

**Affiliations:** Department of Biotechnology, Motilal Nehru National Institute of Technology Allahabad, Prayagraj, India

**Keywords:** visceral leishmaniasis, *Leishmania donovani*, linoleic acid, *Leishmania* derived microvesicles, immune-modulation

## Abstract

Visceral leishmaniasis is a neglected tropical disease caused by *Leishmania (L.) donovani* parasite in the Indian subcontinent. Macrophages (mϕ) are the harboring cells for parasite and their interactions dictate the pathogenesis of this disease. Polyunsaturated fatty acids are an integral part of the mϕ cell membrane and are derived from linoleic acid (LA), which is a principal essential fatty acid. Here, we have investigated the effect of the simultaneous presence of LA during *L. donovani* infection in mϕ. Treatment with LA suppresses the parasitic load in mϕ (kDNA expression) and promotes the Th-1 type immune response (IL-12, iNOS). However, no significant change in kDNA expressions was observed when *L. donovani* promastigotes were treated with LA. Intrigued by this observation, we explored mechanism(s) by which LA promoted the protective type immune response in infected mϕ. Interestingly, LA decreased the release of *L. donovani* derived extracellular vesicle later characterized as microvesicles. Moreover, these microvesicles were suppressive concerning their bias toward the Th-2 type of immune responses (IL-10, Arginase) in mϕ. We suggest that LA plays a protective role in the immune response against *L. donovani* infection by inhibiting the release to *Leishmania* derived microvesicles and thus promoting Th-1 type immune response in mϕ.

## Introduction

*Leishmania (L.) donovani* is the causative agent of disease visceral leishmaniasis (VL) in the Indian subcontinent. The disease affects primarily underprivileged people in Bihar, Jharkhand, West Bengal and eastern part of Uttar Pradesh. The global incidences of VL are between 50,000 and 90,000 (WHO, 2019). There is no human vaccine available against VL in the market and chemotherapy has its own drawbacks especially evolving drug resistance (Mishra et al., 2007). The emergence of post kala-azar dermal leishmaniasis (PKDL) cases in the past few years (NVBDCP, 2019), HIV-VL co-infection (Sinha et al., 2005), the emergence of drug resistance (Mishra et al., 2007) and unavailability of a human vaccine against VL suggest a need for more effective control measures. Advancement in controlling the disease requires an improved understanding of the host-parasite interaction in VL infection.

The pathogenesis of VL depends significantly upon macrophage (mϕ)-*Leishmania* interactions and further their encounter with T cells. As major components of the cellular membrane, polyunsaturated fatty acids (PUFA) play a pivotal role in maintaining the membrane fluidity and increase the innate immune threshold in mϕ, which is essential for appropriate antigen presentation to T cells (Sen et al., 2001). It is evident that mϕ enriched with PUFAs specially arachidonate showed ~50% enhancement of phagocytic and adhesion capacity (Calder et al., 1990). Linoleic acid (LA) is an essential polyunsaturated fatty acid (PUFA) and the precursor of long-chain PUFAs in the mammals. Various groups including ours have reported the role of prostaglandins and leukotrienes in *Leishmania* infection (Morato et al., 2014; Saha et al., 2014; Chaves et al., 2016; Saini et al., 2019). However, supplementation with their dietary precursor i.e., LA is a more viable and feasible approach in the treatment of human VL, but such studies are lacking.

In the present study, we have analyzed the effect of the simultaneous presence of LA during *L. donovani* infection in mϕ. LA suppressed parasitic load (kDNA gene expression) in infected mϕ and promoted Th-1 type protective immune response. However, it did not show any direct leishmanicidal activity *in vitro* on *L. donovani* promastigotes. Further exploration showed that LA reduces the release of extracellular vesicles from *L. donovani* parasite (promastigote form), which were later characterized as microvesicles (Muralidharan-Chari et al., 2010; Raposo and Stoorvoel, 2013; Marcilla et al., 2014). The immunomodulatory properties of these *L. donovani* derived microvesicles (*Ld*Mv) were also analyzed in *vitro* and were found to be immunosuppressive. Taken together, our data indicate that LA modulates the release of *L. donovani* derived microvesicles (*Ld*Mv) that restraint the parasitic load and promote pro-inflammatory type immune response.

## Materials and Methods

### Reagents

RPMI-1640 media (Cat. No. 31800-022, Gibco Life Technologies, USA), FBS (Cat. No. 10270106, Gibco Life Technologies, USA), antibiotic cocktail (Penicillin + Amphotericin B + streptomycin; only for maintenance media; Cat. No. E485, Amresco Inc., USA), Kanamycin (only for infection media; Cat. No. Kanamac-750, Macleod Pharmaceuticals Ltd., India), NaHCO_3_ (Cat. No. 27765, Fischer Scientific Pvt. Ltd., India), HEPES (Cat. No. MB016, Himedia Laboratories Pvt. Ltd., India), and L-Glutamine (Cat. No. G0063, TCI Chemicals Pvt. Ltd., India) were procured for cell culture purpose. Pure Linoleic Acid (LA; Cat. No. sc-200788A) was purchased from Santa Cruz Biotechnology, USA. Details of other reagents are given in their respective method sections.

### Parasite

Promastigote form of *L. donovani* (dd8; MHOM/IN/80/Dd8; WHO reference strain of Indian origin) was maintained in M199 medium with 10% FBS at 25–27°C in anaerobic condition (Tiwari et al., 2016). The parasite strain was obtained from the Central Drug Research Institute, Lucknow, India. These parasites were being routinely maintained in our laboratory (MNNIT Allahabad) and were tested for expressions of *L. donovani* specific actin/tubulin gene regularly. For experiments, cells were fixed with formaldehyde (4%) and counted using a Neubauer chamber.

### Culture of Macrophage Cell Line and *in vitro* Assays

The mouse mϕ cell line (J774A.1) was obtained from Central Drug Research Institute, Lucknow, India and maintained in RPMI-1640 media (with 10% FBS) at 37°C in 5% CO_2_ incubator (Model No. ESCO CelSafe, Esco Micro Pvt. Ltd., Singapore) at MNNIT Allahabad (Saini et al., 2019). For experiments, 2 × 10^6^ mϕ cells were counted with the help of the Neubauer chamber and seeded in the six well plates. After 3–4 hours (h), non-adherent cells were removed and the assay was performed with adhered cells. To establish the infection of *L. donovani* to mϕ, adherent J774A.1 mϕ cells were incubated with the 20 × 10^6^ *L. donovani* parasites (MOI; 1:10). After 12 h of incubation, the non-infecting parasites were removed by washing with sterile PBS/incomplete RPMI (thrice). As per the experimental plan, cells were treated with 500 nM LA in culture (on the basis of literature) along with the parasite infection. After completion of assays, cells were processed for RNA/DNA isolation.

### Gene Expression Analysis Using Real-Time PCR (qPCR)

Total RNA was isolated from mϕ (uninfected as well as infected) and processed further as described by Saini et al. (2020a). All the primers used in the present study (Supplementary Table 1) were commercially synthesized from Eurofins genomics, India. Expression analysis of gene was performed using qPCR (Cat. No. TCR0096, PikoReal real-time PCR System). The housekeeping gene i.e., HGPRT (cell line)/α-tubulin (parasite) was used to normalized cT values in control and experimental tubes. The data is represented as fold change in gene expression (2^−ΔΔcT^ value) considering untreated mϕ as reference/control (fold change = 1) (Schmittgen and Livak, 2008; Sindhu et al., 2018).

### Measurement of Parasite Load Using kDNA

A modified Fan and Gulley method was used for the isolation of DNA from infected mϕ cells (Fan and Gulley, 2001). Briefly, the parasitic load in infected mϕ was quantified by measuring the copy number of *L. donovani* specific kDNA in isolated DNA using qPCR (Verma et al., 2019).

### Fluorimetry Analysis

The culture supernatant of *L. donovani* parasite was incubated with 10 μM Diphenylhexatriene (DPH; Cat. No. 66525, SRL Pvt. Ltd., India) for 60 min at room temperature. After completion of incubation, the analysis was performed in a fluorescence spectrophotometer (LS-45, Perkin Elmer Inc., USA) in the Center for Interdisciplinary Research (CIR), MNNIT Allahabad. Scan between 385 and 650 nm was performed and maxima was observed at 430 nm. Hanks' balanced salt solution (HBSS) (Tiwari et al., 2016) alone was taken as background and was deducted to get normalized fluorescence intensity. Experiments were performed three times independently and every time in triplicate.

### GC-HRMS Analysis

To assess the fatty acid profile of culture supernatant of *Leishmania donovani*, GC-HRMS was used. Fatty acids from culture supernatant were extracted using Bligh and Dyer method (chloroform/methanol method) (Bligh and Dyer, 1959). Briefly, 3.75 ml chloroform and methanol (1:2) were added to 1 ml sample (supernatant). Heptadecanoic acid (5 mM/L) (Cat. No. H3500, Sigma Aldrich, USA) was added as the internal standard. Tubes were vortexed well and 1.25 ml Chloroform was added. After mixing, an equal amount (1.25 ml) of double distilled water was added. Then, the tubes were centrifuged at 1,000 rpm for 5 min at room temperature. The lower organic phase was recovered and the solvent was dried. The lipid extract obtained was dissolved in 2 ml BF_3_-Methanol (14%). Sealed tubes were kept at 55°C for 1.5 h with vigorous shaking after every 20 min. After 1.5 h, 2 ml saturated NaHCO_3_ solution and 2 ml Hexane was added. Tubes were centrifuged at 1,000 rpm for 5 min and the organic phase was collected for analysis. Final samples were transferred to screw cap glass tubes and were transported to SAIF- IIT Bombay for GC-HRMS analysis under appropriate conditions.

### Characterization of Extracellular Vesicles Released by *L. donovani (Ld)* Promastigotes

5 × 10^6^/ml parasites were cultured for 6 h with and without LA (significant uptake of LA was observed by promastigotes form of parasite after 6 h). After 6 h, the supernatants were harvested after centrifugation at 1,200 rpm for 10 min to remove parasites. These stationary phase *L. donovani* promastigotes were washed with PBS and fixed in 2.5% glutaraldehyde overnight (25°C). Fixed parasites were washed, dissolved in sterile water, placed on glass coverslips and kept at 37°C for complete drying. The supernatant was further centrifuged twice at 10,000 rpm for 30 min to harvest secreted microvesicles (Mv) (Greening et al., 2015). These Mv and dried parasite samples were transported to All India Institute of Medical Sciences (AIIMS) New Delhi for further analysis. Samples were analyzed under the Scanning Electron Microscope (EVO18 Zeiss, Oberkochen, Germany) in the sophisticated analytical instrumentation facility of AIIMS New Delhi. Besides this, the isolated Mv was finally dissolved in PBS and analyzed in particle size analyzer (Nanotrac Wave, Microtrac, USA) in CIR, MNNIT Allahabad.

### Statistical Analysis

Experiments were performed independently in three sets. Each reaction was carried out in duplicates. The data are represented as Mean ± S. D. To compare the differences between two groups, student's *t*-test (paired and unpaired) was used. A *p* < 0.05 was regarded as significant and shown with the graph. The analysis was done using SPSS 15.0 and Graph Pad Prism-5.0. All the graphs and figures were made using Graph Pad Prism-5.0.

## Results

### LA Decreases the Parasitic Load in mϕ and Promotes Pro-inflammatory Response

**Figure 1:**
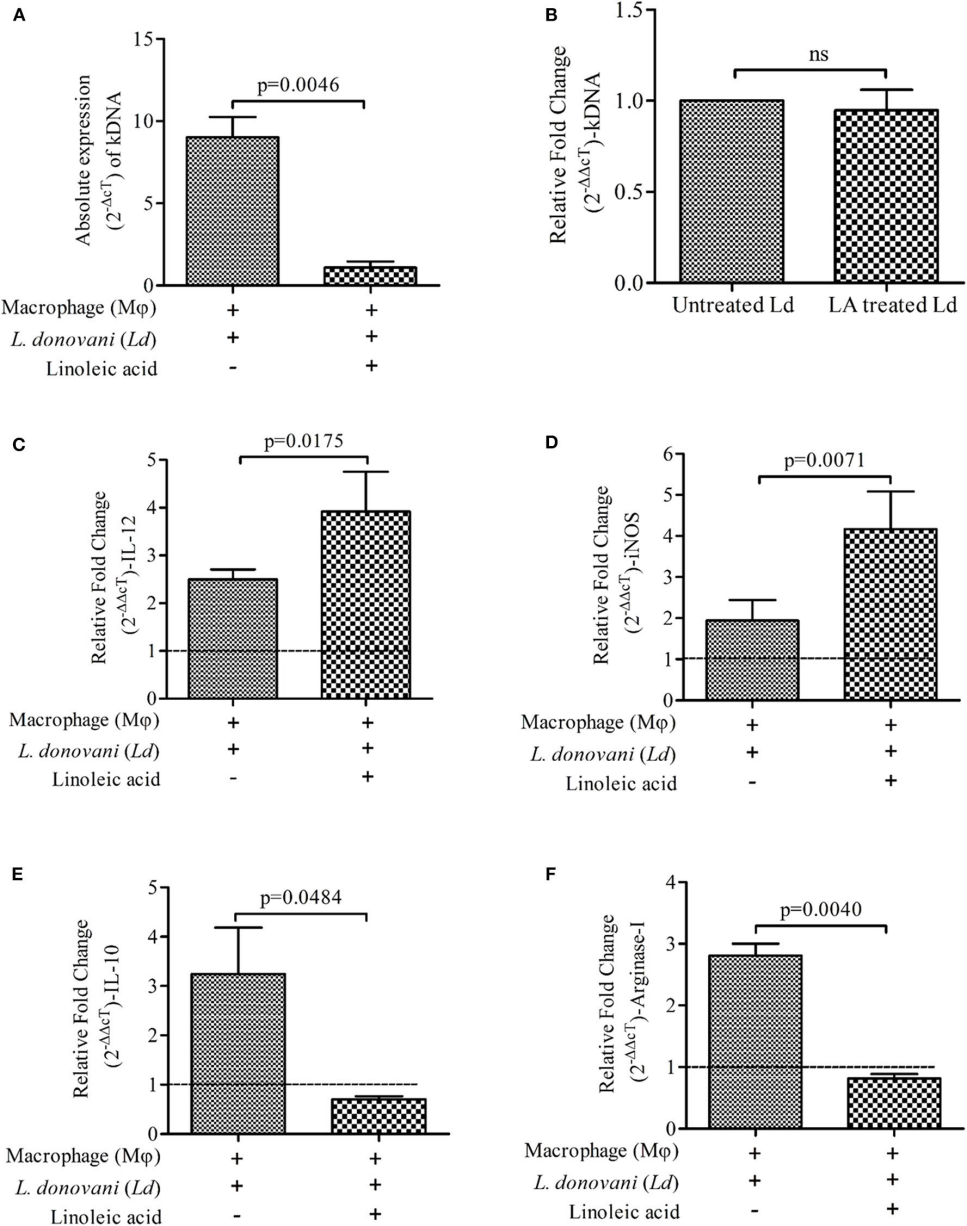
Presence of Linoleic acid (LA) at the time of *Leishmania donovani (Ld)* infection to macrophages (mϕ) decreases the parasite load and modulates the immune response. **(A)** The absolute expression (2^−ΔcT^) of kDNA i.e., parasite load, is shown in *Ld* infected mϕ with and without supplementation of LA. The treatment of LA has been given simultaneously at the time of infection. **(B)** The bar diagram shows the relative expressions (2^−ΔΔcT^) of kDNA in *Ld* promastigotes upon treatment with LA. Relative fold change (2^−ΔΔcT^) in mRNA expressions of IL-12 **(C)**, iNOS **(D)**, IL-10 **(E)**, and Arginase-I **(F)** being shown under three different conditions i.e., mϕ (J774A.1) alone (uninfected control; horizontal dotted line, fold change = 1), *Ld* infected mϕ and *Ld* infected mϕ + LA. The results are representative of three independent experiments and each experiment was performed in triplicate. Data are expressed as Mean ± S. D. and significant differences are shown as the *p*-value on the graph.

Presence of LA at the time of *L. donovani* infection to mϕ suppresses the parasite load in infected mϕ (Figure 1A; Mean ± S. D., 1.092 ± 0.3801) as compared to infected control (infected mϕ without any treatment) (Mean ± S. D., 9.018 ± 1.243) (Figure 1A). However, we didn't observe any significant change in kDNA of *L. donovani* promastigotes upon treatment with LA (without mϕ; Figure 1B). Findings showed increased expressions of immune markers of Th-1 type upon LA treatment in *L. donovani* infected mϕ [IL-12 (Figure 1C; Mean ± S. D., 3.917 ± 0.833) and iNOS (Figure 1D; Mean ± S. D., 4.167 ± 0.917)] as compared to control (IL-12; Mean ± S. D., 2.497 ± 0.209 and iNOS; Mean ± S. D., 1.945 ± 0.497). Treatment of LA also showed a simultaneous decrease in the Th-2 type immune markers in *L. donovani* infected mϕ [IL-10 (Figure 1E; Mean ± S. D., 0.7025 ± 0.0638) and Arginase-I (Figure 1F; Mean ± S. D., 0.816 ± 0.070)] as compared to infected but untreated mϕ (IL-10; Mean ± S. D., 3.243 ± 0.942 and Arginase-I; Mean ± S. D., 2.807 ± 0.195). For calculation of relative fold change in expressions, uninfected mϕ were taken as control (fold change = 1, shown as a dashed line in figures). The significant difference between two groups was analyzed using paired *t*-test.

### LA Reduced the Release of Membranous Bodies in the Supernatant of *L. donovani* Culture

**Figure 2:**
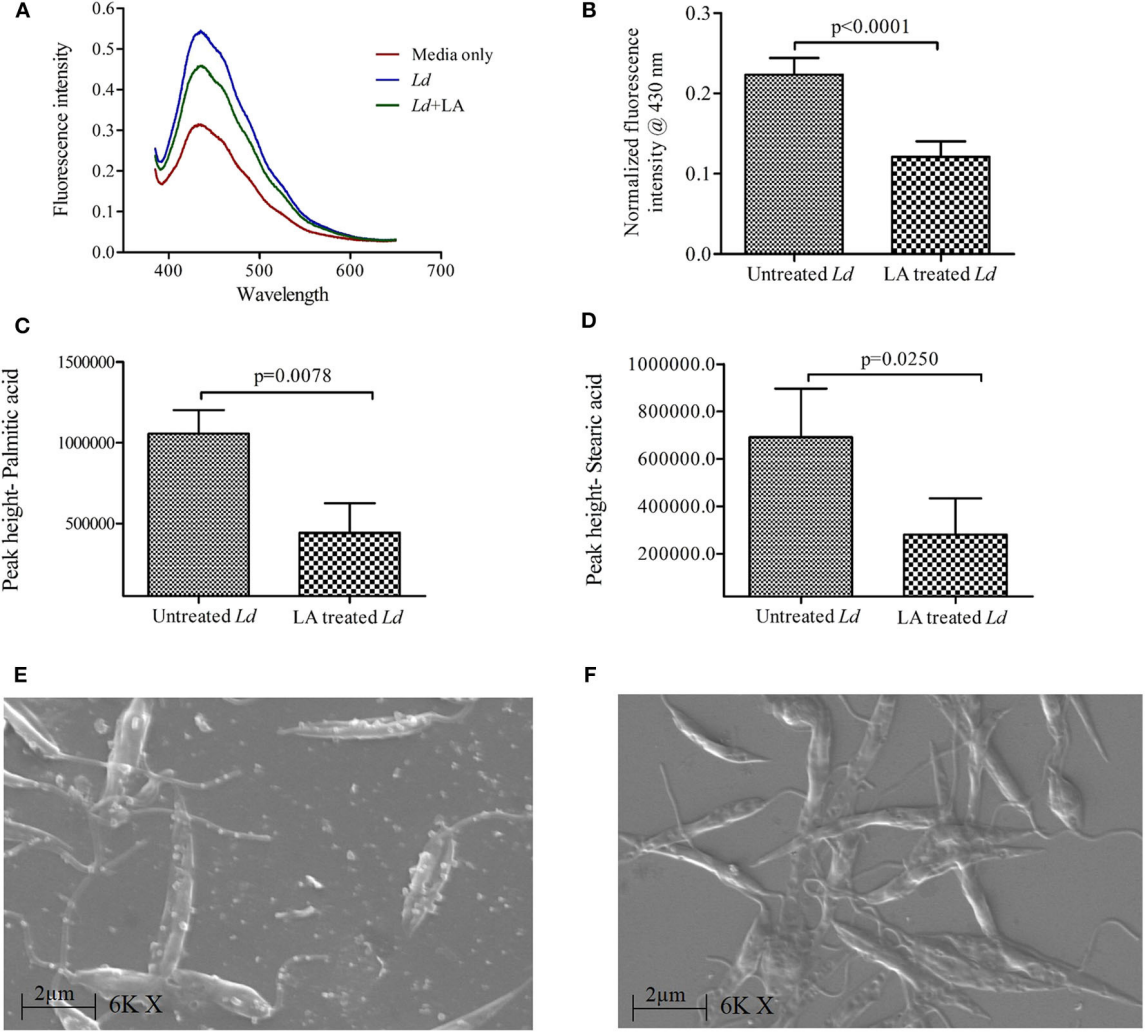
Release of extracellular membranous bodies in the culture supernatant of *Leishmania donovani* (*Ld*) promastigote. **(A)** Fluorescence intensity spectra of culture supernatants of *Ld* promastigotes are shown using diphenylhexatriene dye (DPH, 10 μM) under three conditions i.e., HBSS media only, *Ld* culture and *Ld* culture treated with linoleic acid (LA). **(B)** The cumulative findings of fluorescence intensity (at 430 nm) are shown where culture supernatants of *Ld* (LA treated and untreated) were incubated with DPH dye. The intensities in experimental conditions were first normalized using that of HBSS media only. Bar diagram shows the differences in levels (peak height) of **(C)** palmitic acid and **(D)** stearic acid in culture supernatants of untreated and LA treated *Ld* promastigotes using GC-HRMS. **(E,F)** The scanning electron micrographs of *Ld* promastigotes are shown in two different conditions i.e., untreated and LA treated, respectively. Magnification is 6,000× with 2 μm scale and EHT 20.00 kV. Experiments were performed three times independently in triplicates and significant differences (*paired t-test*) are shown as the *p*-value on the graph.

Supernatants of *L. donovani* promastigote cultures (LA treated and untreated) were analyzed for the presence of membrane bodies using diphenylhexatriene (DPH) dye (10 μM) (Figures 2A,B). DPH dye binds to cell membranes and demonstrates strong fluorescence when intercalated into lipid membranes. We have observed high fluorescence intensity of DPH in the supernatant of untreated culture (Mean ± S. D., 0.223 ± 0.020) as compared LA treated culture (Mean ± S. D., 0.121 ± 0.019) (Figure 2B), which suggests a low number of membranous bodies in treated samples.

The presence of fatty acid (FA) in the culture supernatant of promastigote culture was analyzed by GC-HRMS, as it also indicates the presence of membranous bodies (Chromatograph- Supplementary Figure 1). Internal standard (Heptadecanoic acid) was identified at 15.4 min. GC-HRMS results revealed that the concentrations of Palmitic acid (PA, 16:0; 14.3 min) and Stearic acid (SA, 18:0; 16.5 min) were decreased in LA treated *L. donovani* culture supernatant (Mean ± S. D., 442,600 ± 182,800 and 20,800 ± 153,200, respectively) as compared to untreated samples (Figures 2C,D; Mean ± S. D., 1,056,000 ± 141,600 and 691,600 ± 205,000, respectively). Hence, our GC-HRMS data also points toward the presence of membranous bodies in the *L. donovani* promastigote culture supernatant, which are decreased upon LA treatment.

**Figure 3:**
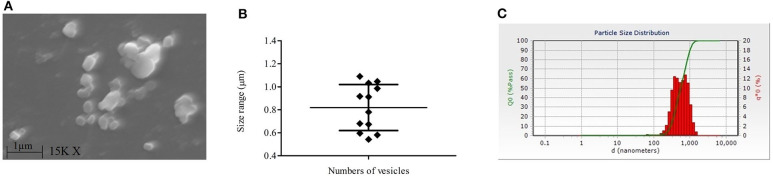
Characterization of extracellular vesicles released by *L. donovani (Ld)* promastigotes. **(A)** Image of scanning electron microscopy (SEM) shows the presence of extracellular vesicles in the culture supernatant of the *Ld* parasite. **(B)** Sizes of these vesicles in SEM analysis are shown and it ranges from 0.5 to 1 μm in size (Mean ± S. D., 0.819 ± 0.200). **(C)** Findings of particle size analysis are shown where culture supernatants of *Ld* culture were checked for particles' sizes. Experiments were performed three times independently in triplicates.

Our SEM data showed the release of membranous bodies/extracellular vesicles by *L. donovani* promastigotes under appropriate culture conditions, which was characterized by vesicular blebbing of the cell membrane (Figure 2E). The same was not observed upon treatment with LA (Figure 2F). To characterize whether these membranous bodies are exosomes (30–100 nm) or/and microvesicles (0.1–1 μm) (Marcilla et al., 2014), excreted membranous bodies were characterized using SEM and particle size analysis (PSA). The findings of SEM showed the presence of vesicles of sizes ranging from 0.5 to 1 μm in size (Figures 3A,B; Mean ± S. D., 0.819 ± 0.200 μm) which characterize these vesicles as microvesicles (Muralidharan-Chari et al., 2010; Raposo and Stoorvoel, 2013; Marcilla et al., 2014). Results obtained from PSA also suggested the same and the average size of vesicles was also ranging between 0.5 and 1 μm (Figure 3C; Mean ± S. D., 0.729 ± 0.269 μm).

### Immuno-Modulatory Properties of *L. donovani* Derived Microvesicles (*Ld*Mv)

**Figure 4:**
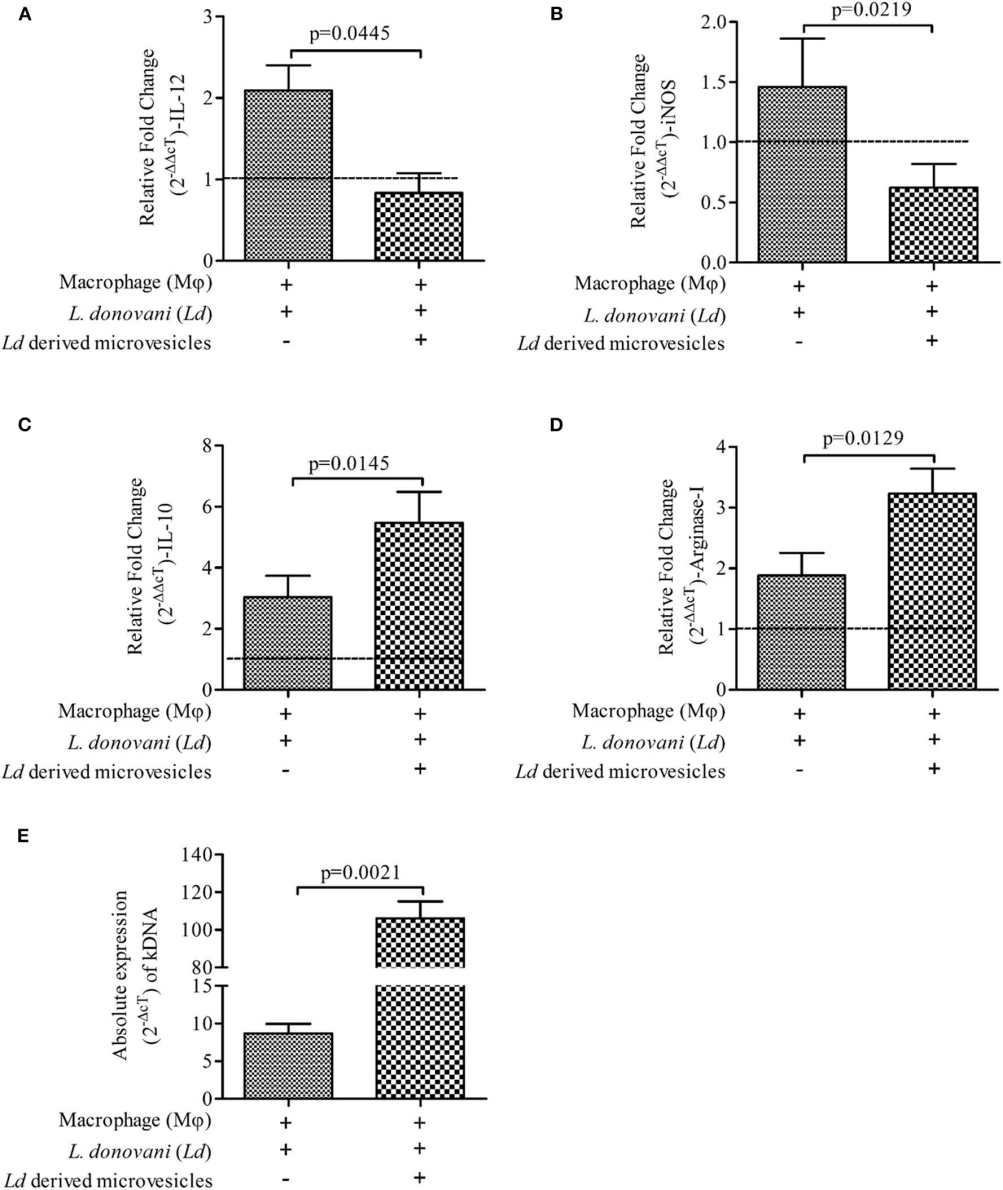
Suppressive nature of *Leishmania donovani* (*Ld*) derived microvesicles (*Ld*Mv). Relative fold change (2^−ΔΔcT^) in the expressions of mRNA of **(A)** IL-12, **(B)** iNOS, **(C)** IL-10, and **(D)** Arginase-I genes are shown in three different conditions i.e., (i) mϕ (J774A.1) alone (uninfected control; horizontal line, fold change = 1), (ii) *Ld* infected mϕ, and (iii) *Ld* infected mϕ + *Ld*Mv. **(E)** Absolute levels (2^−ΔcT^) of kDNA are shown in two conditions i.e., (i) *Ld* infected mϕ (infected control) and (ii) *Ld* infected mϕ + *Ld*Mv. The results are representative of three independent experiments and each experiment was performed in triplicate. Data are expressed as Mean ± S. D. and significant differences are shown as the *p*-value on the graph.

Intrigued by our observation, we were interested to understand the immunomodulatory properties of *Leishmania donovani* derived microvesicles (*Ld*Mv) and its effects on parasite load within mϕ. *Ld*Mv were first generated from 5 × 10^6^/ml of parasite culture and used in *in vitro* experiments as per the plan. In the presence of *Ld*Mv, *Ld* infected mϕ showed decreased expressions of pro-inflammatory immune markers [IL-12 (Figure 4A; Mean ± S. D., 0.834 ± 0.242) and iNOS (Figure 4B; Mean ± S. D., 0.622 ± 0.198)] as compared to *Ld* infected mϕ without treatment (infected mϕ only) (IL-12: Mean ± S. D., 2.095 ± 0.305 and iNOS: Mean ± S. D., 1.458 ± 0.404). Simultaneously, expressions of anti-inflammatory markers (IL-10 and Arginase-I) were increased after *Ld*Mv treatment [IL-10 (Figure 4C; Mean ± S. D., 5.470 ± 1.011) and Arginase-I (Figure 4D; Mean ± S. D., 3.236 ± 0.405)] as compared to infected control (IL-10: Mean ± S. D., 2.036 ± 0.700 and Arginase-I: Mean ± S. D., 1.886 ± 0.370). Uninfected mϕ (i.e., without *Ld* infection) were taken as control (fold change = 1, *dashed line*). Interestingly, we observed an increase in parasite load (absolute gene expression; 2^−ΔcT^) when mϕ were infected with *Ld* + *Ld*Mv (Figure 4E; Mean ± S. D., 8.696 ± 1.254) as compared to only infected mϕ as control (Mean ± S. D., 106.2 ± 8.921). This should also be noted that culture supernatant without *Ld*Mv (from LA treated culture supernatant) has no effect on immune response against *Ld* infection (Supplementary Figure 2).

## Discussion

At the cellular level, the deficiency of LA i.e., ω-6 PUFA, an essential fatty acid, impairs cell-to-cell interaction by modifying cell adhesion (Jianga et al., 2000) and possibly leading to the poor synapse formation and thus compromised activation and antigen presentation (Courtney et al., 2003). LA is the dietary precursor of arachidonic acid (AA) and precursor of the long-chain PUFAs in the mammals. It is evident that mϕ enriched with AA showed ~50% enhancement of phagocytic and adhesion capacity (Calder et al., 1990). AA gives rise to various bioactive molecules e.g., prostaglandins (PGs) and leukotrienes (LTs). Various groups have emphasized on roles of PGs and LTs in the immune response against *Leishmania* infection (Morato et al., 2014; Saha et al., 2014; Chaves et al., 2016; Saini et al., 2019). However, the possibilities of their therapeutic applications are limited, as these molecules are transient in nature as well as not cost-effective. Instead of them, using its dietary precursor i.e., LA may have a beneficial role in the containment of the disease. Our previous observation showed that the preventive, as well as therapeutic usage of LA, significantly contains the parasite load in infected mϕ (Saini et al., 2020b). Before taking these leads further, we were interested to observe the establishment of *Ld* infection in mϕ under LA sufficient condition.

In the present study, LA enriched macrophages successfully eliminated *Ld* infection. We measured the expressions of pro- and anti-inflammatory markers in LA treated and *Ld* infected mϕ. Our finding clearly showed the strengthening of pro-inflammatory and weakening of anti-inflammatory markers, when *Ld* infection was being established in presence of LA (Figure 1). We conclusively demonstrated that the parasite load (i.e., kDNA expression) was also decreased under the same condition, suggesting the poor establishment of infection of *L. donovani* in an LA sufficient condition. However, we failed to observe the killing of *Ld* promastigotes when treated with LA. Summarily, there is no direct leishmanicidal activity of LA and the observed decrease in parasite load in LA treated infected mϕ is possibly due to protective switching from anti- to pro-inflammatory type of immune response. Similar results were obtained when LA was given to either prior to *Ld* infection (preventive) or after *Ld* infection (therapeutic) (Saini et al., 2020b).

**Figure 5:**
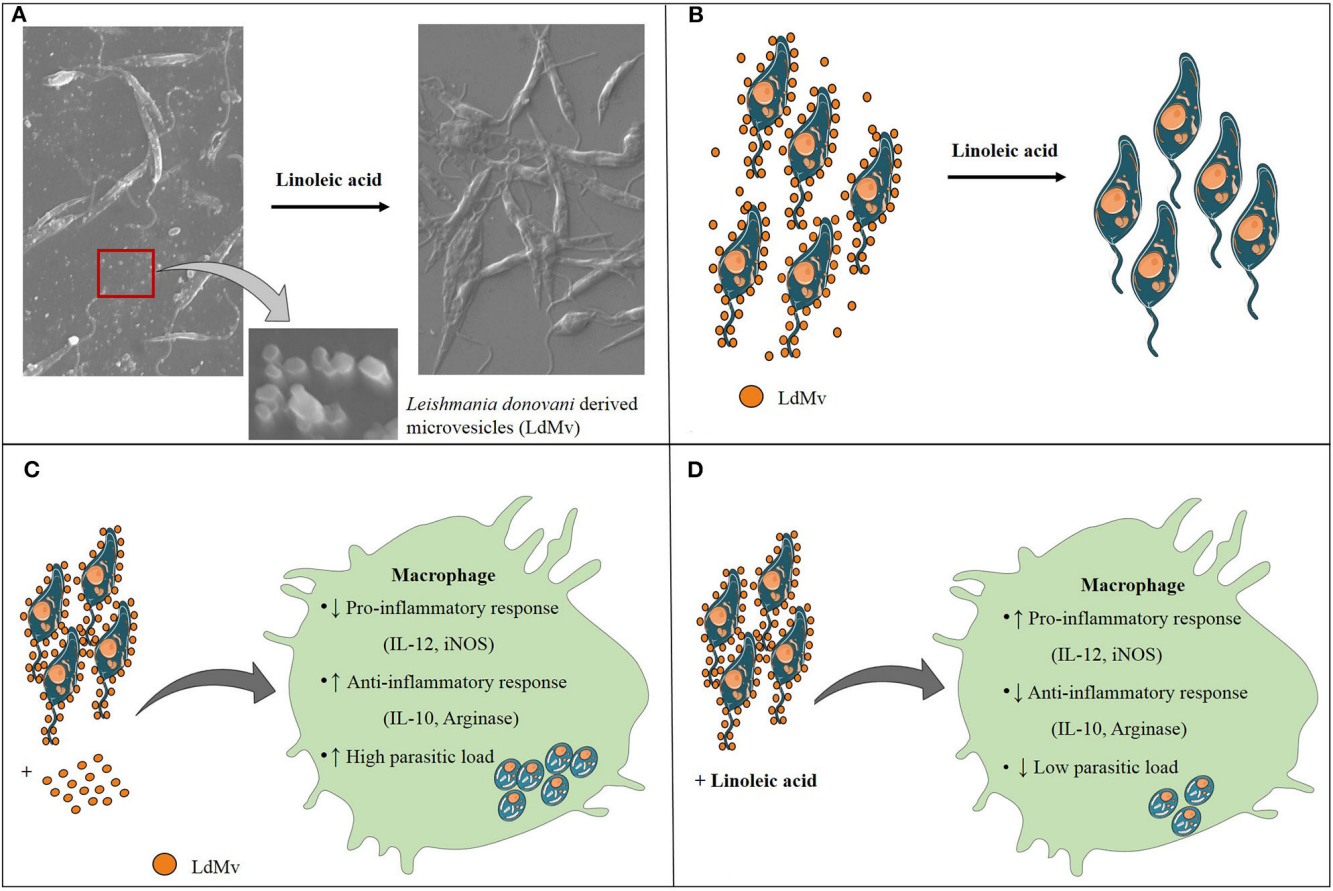
Conclusion of the study. **(A)** Leishmania donovani (Ld) releases microvesicles (Mv), which are suppressed in the presence of linoleic acid. **(B)** Schematic diagram of LdMv inhibition by linoleic acid. **(C)** The infection of *Ld* in the presence of *Ld*Mv (exogenous) suppressed the protective response and up-regulated the inhibitory response, which helped in the dissemination of infection. **(D)** On the other hand, situation was reversed in the presence of linoleic acid which led to the restriction of *Ld* infection.

Our findings suggest the release of membranous bodies in the *Ld* culture, which is clearly visible on the surface of the parasite in SEM i.e., vesicular blebbing. These bodies which are released under normal culture conditions gets inhibited in the presence of LA and these categorically belong to microvesicles (0.5–1.0 μm) (Muralidharan-Chari et al., 2010; Raposo and Stoorvoel, 2013; Marcilla et al., 2014). The presence of palmitic and stearic acid in the culture supernatant of parasite suggest the same and these fatty acids contribute to ~98% of phospholipids (constituents of the cell membrane) in *L. donovani* promastigotes (Messaoud et al., 2017). Szempruch et al. (2016) summarizes the immunomodulatory activities of membranous bodies by protozoan parasites. Previous studies show the release of exosomal membranous bodies by amastigotes form of *Leishmania* parasite and their immunomodulatory properties are also shown. Silverman et al. (2009) proposed an exosome-based pathway from the *Leishmania* parasite that is responsible for protein secretion and communication with mϕ. *L. donovani* exosomes are known to modulate human monocyte cytokine responses by promoting Th-2 type immune response (IL-10) and inhibiting Th-1 type immune response (TNF-α, IL-12p70). Simultaneously, BALB/c mice exposed to *L. major* exosomes showed increased IL-4 production and decreased IFN-γ production at disease sites (spleen and draining lymph node) and exacerbation of *L. major* infection (Silverman et al., 2010). Hassani (2013) provide evidence about protein release from *L. mexicana* via exovesicles (40–100 nm, size range of exosome) during early moments of interaction with the mammalian host in order to that modulate signaling and functioning of the mϕ. It was further showed that GP63 bearing *L. major* exosomes have inflammatory properties and are capable of immune modulation at both signaling and gene expression levels in mϕ (Hassani et al., 2014). All these studies focused on exosomes, however other types of secreted extracellular vesicles like microvesicles (up to 1 μm) remain unexplored. Our study is the first of its kind which suggests the immunomodulatory activity of *Ld* secreted microvesicle (*Ld*Mv). Our findings indicated that the treatment with *Ld*Mv tilts the immune response toward M-1 type (IL-12 ↑ and iNOS ↑; IL-10 ↓ and Arginase-I ↓). Possibly, the release of *Ld*Mv tunes the mϕ and sets a conducive platform (Th-2 ↑) in the mϕ for the establishment of infection (Figure 4). This must also be noted that culture supernatant without *Ld*Mv had no effect on immune response against *Ld* infection (Supplementary Figure 2). Not only in the human host, but *Leishmania* parasite also secretes exosomes in the midgut of sand fly and are the part of sandfly's infective inoculum (Atayde et al., 2015). However, the release of Mv by the parasite and its role in the establishment of infection was not yet established. To the best of our knowledge, our study first time demonstrates the release and role of *Leishmania donovani* promastigote derived microvesicles in the establishment of infection. Moreover, its release is inhibited by the presence of LA i.e., a nourished condition, in the culture. We are suggesting that LA plays a dual role in the protective immune response against VL infection. It decreases the release of *Ld*Mv from the promastigote form of *Leishmania* parasite and promotes the pro-inflammatory response inside mϕ via the 5-lipoxygenase pathway to eliminate parasite present inside. This study highlights the immunomodulatory properties of *Ld*Mv and the possible role of nutrient i.e., LA in inhibiting their release which leads to the containment of *L. donovani* infection (Figure 5). Conclusively, it is believed that *Leishmania* secreted microvesicles exert modulation of immune responses.

## Data Availability Statement

The datasets generated for this study are available on request to the corresponding author.

## Author Contributions

SS performed all the experiments, analyzed as well as interpreted the data, and wrote the manuscript. AR conceptualized, designed the work, managed funding, and edited the manuscript. All authors contributed to the article and approved the submitted version.

## Conflict of Interest

The authors declare that the research was conducted in the absence of any commercial or financial relationships that could be construed as a potential conflict of interest.
