# Supplementary material for: Linoleic Acid Inhibits the Release of Leishmania donovani Derived Microvesicles and Decreases Its Survival in Macrophages
Source: Front Cell Infect Microbiol. 2020 Aug 7;10:406. doi: 10.3389/fcimb.2020.00406 (PMC7426612; doi:10.3389/fcimb.2020.00406)
Supplement: Supplementary file 1 [file Table_1.DOCX]

**Supplementary online information**

Table 1- Details of primers used in the present study.

| **Primer name** | **Accession Id** | **Primer sequences** |
| --- | --- | --- |
| HGPRT | NM_013556.2 | FP-5’-GTTGGGCT TACCTCACTGCT-3’  RP-5’-TAATCACGACGCTGGGACTG-3’ |
| IL-12 | M86671.1 | FP-5’-TTGCCATCGTTTTGCTGGTG-3’  RP-5’-GGTCAGGGTCTTTCCAGAGC-3’ |
| IL-10 | NM_010548.2 | FP-5′-ACCTGCAGTGTGTATTGAGTCTG-3′  RP-5′-CCCTGGATCAGATTTAGAGAGC-3′ |
| iNOS | XM_006532446.3 | FP-5’CTTTGCCACGGACGAGAC-3′  RP-5’TCATTGTACTCTGAGGGCTGAC-3’ |
| Arginase-I | NM_001281645.1 | FP-5’- CACTGGGAATTTGCATGGGC-3’  RP-5’-GGTCTACATCTCGCAAGCCA-3’ |
| α-tubulin | XM_003859197.1 | FP-5’-ACATCACGAACTCGGTGTTT-3’  RP-5’- TTCGTCTTGATCGTCGCAAT-3’ |
| kDNA | Z35275.1 | FP- 5’-CTTTTCTGGTCCTCCGGGTAGG-3’  RP- 5’-CCACCCGGCCCTATTTTACACCAA-3’ |

**Figure 1**


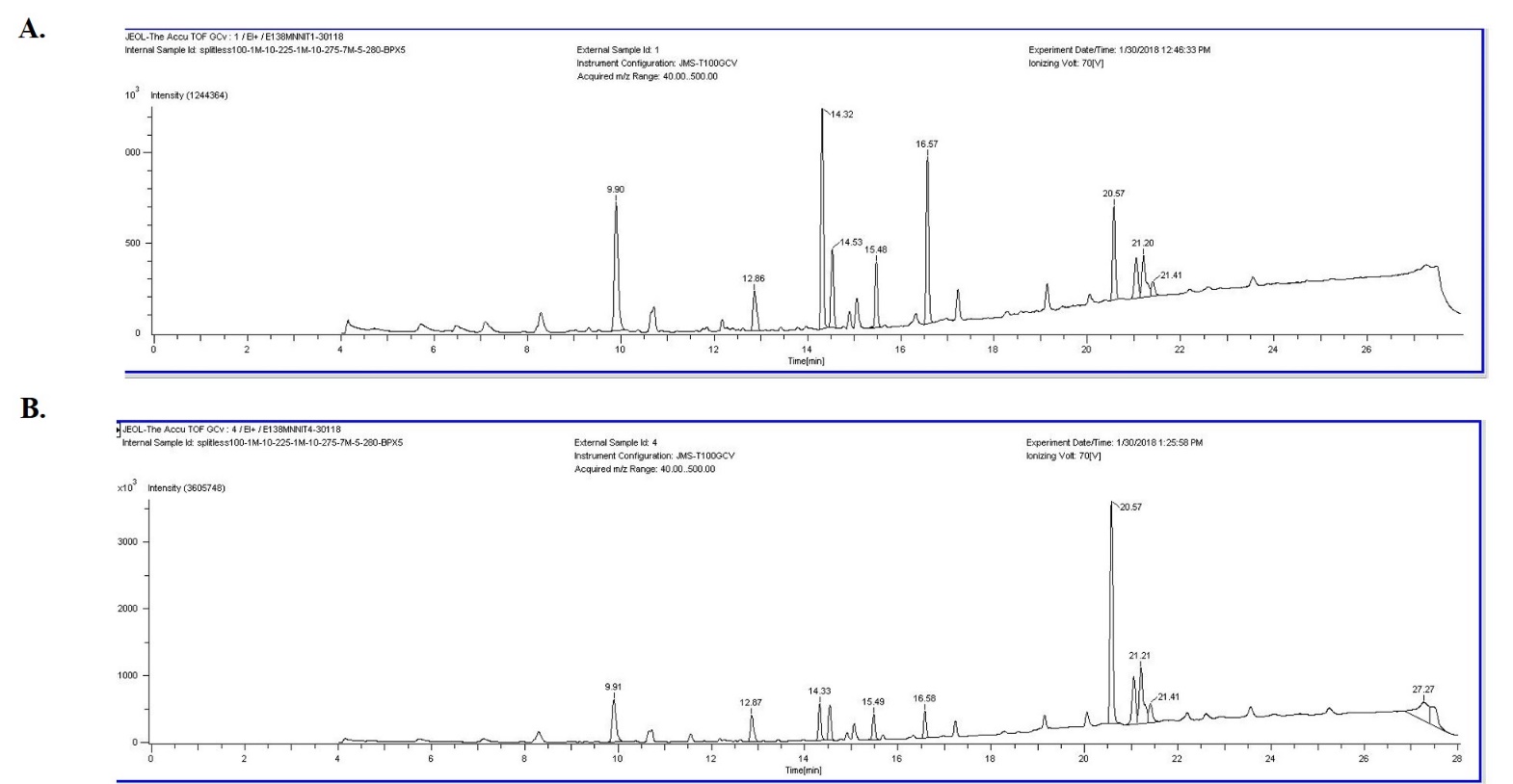


**Figure 1- Chromatograph of GC-HRMS.** The image shows chromatograph (GC-HRMS) of various fatty acids in the culture supernatant of *Leishmania donovani* with **(B)** and without **(A)** linoleic acid.

**Figure 2**


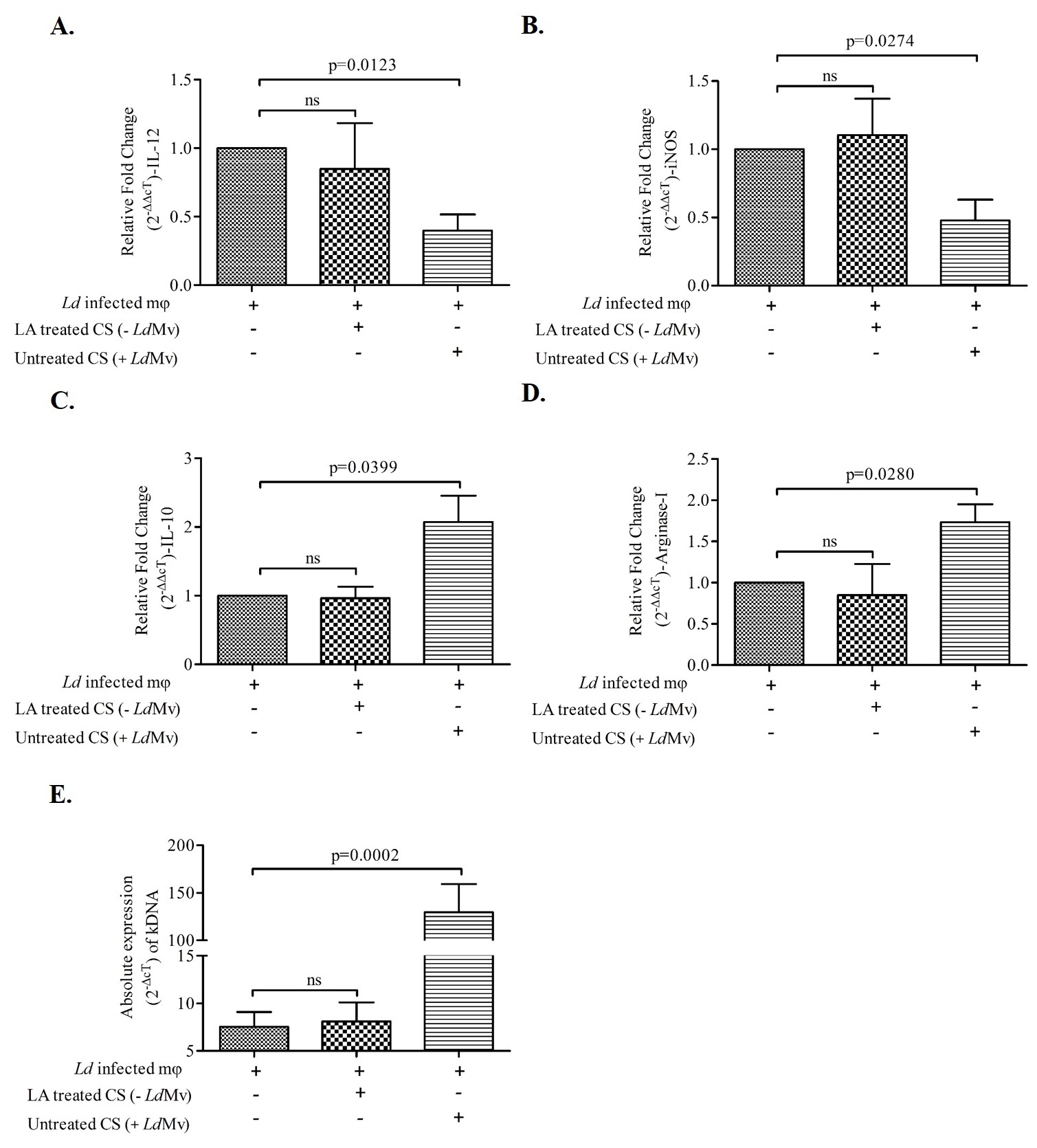


**Figure 2. Immune modulatory effected mediated by *Leishmania donovani* derived microvesicles (*Ld*Mv).** Bar diagram shows the changes in mRNA expressions of **(A)** IL-12, **(B)** iNOS, **(C)** IL-10 and **(D)** Arginase-I genes under three different conditions i.e. *L. donovani*(*Ld*) infected mφ (J774A.1), *Ld* infected mφ + culture supernatant (CS) without *Ld*Mv and *Ld* infected mφ + culture supernatant (CS) with *Ld*Mv. The y-axis shows relative fold change (2^-∆∆cT^) in the expressions of respective genes and *Ld* infected mφ shows the calibrated expression (fold change=1) of the respective gene. The absolute expression (2^−ΔcT^) of kDNA **(E)** shows differences in parasitic load. The results are representative of three independent experiments and each experiment was performed in triplicate. Data are expressed as mean ± S.D. and significant differences are shown as the p value on the graph. ns- non-significant
